# Clinical Memoranda from the Surgical Clinic at the Sisters’ of Charity Hospital

**Published:** 1893-12

**Authors:** Herman Mynter

**Affiliations:** Professor of Surgery, Niagara University, and Surgeon to the Sisters’ Hospital


### Clinical Reports.

### CLINICAL MEMORANDA FROM THE SURGICAL CLINIC AT THE SISTERS OF CHARITY HOSPITAL.

By HERMAN MYNTER, M. D.,
Professor of Surgery, Niagara University, and Surgeon to the Sisters' Hospital.

In a paper on Arthrectomy of the Knee-joint, read before the Medical Association of Central New York, on June 2, 1891, I discussed the importance of the early discovery and prompt removal of the local tuberculous focus in the epiphysis, before the joint had become permanently injured by perforation of the focus directly into the joint, or by extension of the tuberculous process through the periosteum and synovialis. The main symptoms of a local focus, in its early stage, near the epiphyseal cartilage, are diminished extreme extension and flexion, while the motion is free in the middle ranges, slight atrophy and commencing muscular contractions, starting pains, increased surface temperature and pain on tapping over a circumscribed area. Where such symptoms are present, any delay is dangerous, and painting with tincture of iodine, the universal remedy for obscure bone and joint affections, is worse than useless. There is but one thing to do, *i. e.*, to incise down to the bone over the painful area, trephine the epiphysis, and remove the local focus with a sharp spoon. The quickness with which these patients recover by this treatment is as astonishing as it is gratifying. I do not know of anything that gives me more pleasure than the early discovery and removal of such a local focus. The years of sufferings, with resections and amputations as a final resort, which I have saved these unfortunate patients, are quite large. The same symptoms may accompany a chronic osteomyelitis in the diaphysis of the long bones, particularly in those bones which, as in the hip and shoulder joints, have the epiphysis completely surrounded with the synovial capsule. The original pathological condition, the chronic osteomyelitis, is often overlooked, and the patients treated for joint rheumatism or tuberculous joint affections. Yet, a careful examination would show that the joint is perfectly free in the middle ranges and not tender by crowding the joint surfaces together, while the upper or lower third of the diaphysis is thickened, tender on pressure, and doughy in consistency. The accompanying muscular atrophy and diminished motion simply show that the osteomyelitic process has extended near the joint, and that this prepares itself for the inevitable perforation by becoming more or less obliterated. To illustrate these points, I take the liberty to publish a few of the quite numerous cases I have seen :

### TUBERCULOUS EPIPHYSITIS OF TIBIA.

CASE IV.—Alice Goodrich, aged 7 years, entered the hospital on August 18, 1893, with the following history : she sprained her left knee by a fall on the ice, last Winter, but recovered perfectly. A few weeks ago her mother noticed that she limped and could not extend the left leg completely. She became peevish and fretful, and cried at night. At the examination the left knee was seen somewhat swollen, on account of moderate hydarthrus. A distinct doughy swelling was seen over the internal condyle of the tibia, with increased surface temperature and great tenderness on tapping and pressure. Motion in the middle ranges of the joint was free and painless, but forced extension and flexion was very painful. Some atrophy of calf and contraction of hamstring muscles apparent.

A curved incision was made over the internal condyle of the tibia, the periosteum elevated, and a small opening discovered in the bone, through which tuberculous granulations were sprouting out. The opening was enlarged by chisel, and a cavity discovered as large as a hickory nut, and filled with bone detritus and tuberculous granulations. The cavity extended up near the joint cartilage. It was scraped and packed with iodoform gauze under an antiseptic bandage.

August 22d, patient discharged to her home. The cavity was kept open for four weeks, and then healed rapidly. The hydarthrus disappeared in short order, the motions in the knee became free and normal, and she is now in perfect health.

### ACUTE OSTEITIS OF INTERNAL CONDYLE OF FEMUR.

CASE V.—William Garling, aged 20, Walcottsville, N. Y., entered the hospital on May 23, 1893, on crutches, and unable to use his right leg. He was struck on right knee by a horse three weeks previously. There was seen considerable swelling and apparent expansion of internal condyle of right femur, with increased local temperature and pain by pressure and tapping. Complete extension and flexion of the knee-joint was impossible, but motion in middle range was painless. Under narcosis, the internal condyle of the femur was trephined. The periosteum was found thickened, and easily peeled off. The condyle was found in a state of softening and congestion, without any distinct cavity. The osteoporotic bone-tissue was gouged out, leaving a healthy cavity as large as a small walnut. The cavity was plugged with iodoform-gauze, under an antiseptic bandage.

June 4th, wound dressed; looks healthy. Patient can bear considerable weight on the foot.

June 10th, patient discharged. He can walk without crutches, and has no pain in the knee, which appears normal as far as motions are concerned, but he presents a distinct genu-valgum on the right side, evidently the result of the enlargement of the internal condyle on account of the acute inflammation.

TUBERCULOUS OSTEOMYELITIS OF HUMERUS.

Case VI.—William D., aged 27, Hammondsport, N. Y., clerk, entered the hospital on September 20, 1893, with the following history: three years ago the patient had a severe attack of gonorrheal rheumatism, from which he recovered. He has complained for one year of pains under right shoulder, expectoration, cough, and some night-sweat, and has lost considerably in weight.

Three weeks ago he noticed a slight pain in the right shoulder, which has since steadily increased. He describes it as a dull, aching pain, continuing day and night, and the shoulder feels as if there was a heavy weight resting on it. He has been feverish since, lost his appetite, and had night-sweats. He has been treated at home for inflammatory rheumatism with salicylates, but without any benefit.

On examination, he looks pale and suffering. The examination of the lungs does not reveal anything abnormal. There is a diffuse swelling of the upper third of the humerus, while the shoulder-joint itself seems rather smaller and the acromion more prominent than normal, on account of atrophy of the deltoid muscle. Limited motions of the joint and crowding the joint surfaces together are not painful, but raising and rotating the arm are very painful. The skin is reddish, the surface temperature increased, and the humerus, in its upper fourth, feels thickened and is very painful upon pressure. Temperature, 100; pulse, 96. The diagnosis in this case was so evidently that of a bone affection in the neighborhood of the shoulder joints, that I decided on immediate operation. Under narcosis, an incision, four inches long, was made along the anterior margin of the deltoid muscle. Under the muscle a collection of tuberculous granulations was found, but the bone seemed normal here. Another incision was then made along the posterior margin of the deltoid, where the same tuberculous granulations were found. The periosteum was here thickened, and easily peeled off. The medullary cavity of the humerus was chiseled open, just below tuberculum major, and found in a state of tuberculous degeneration, with softening and numerous foci of tuberculous material and small pus-cavities. The disease extended into the head of the humerus, which was gouged out with a sharp spoon, and down the shaft for about three inches. The joint was not opened. The cavity was plugged with iodoform-gauze, the anterior incision closed by sutures, and an antiseptic bandage applied.

He improved very rapidly, the wound was dressed once a week in the same manner, and he left the hospital for treatment in his home, October 5th. October 25th his wounds were all healed ; there was still some stiffness in the joints, for which passive motions were recommended. He had gained twenty-five pounds in weight, and was in perfect health.

### OSTEO-ARTHROITIS HUMERI AND RESECTIO HUMERI.

Case VII.—Mrs. Murphy, 66 years of age, residing at Lockport, N. Y., entered the hospital on June 20, 1893. She had complained for five years of rheumatism in her left shoulder ; eighteen months ago she noticed a slight stiffness and pain in the left shoulder. It grew worse gradually, and interfered greatly with her work. She was treated by various physicians for rheumatism, but without any benefit. About one year ago, the pain became more severe and of a dull, aching character, and the movements of the shoulder became very limited. Blisters, liniments, and anti-rheumatic remedies were again tried unsuccessfully. At last, the joint was aspirated and a turbid fluid removed. Later on, two incisions were made and a similar fluid evacuated. The incisions made did not heal, and continued to discharge a thin, watery fluid. Worn out by pain, she entered the hospital on June 20, 1893.

On examination, the region of the left shoulder was seen uniformly enlarged and swollen, the muscles, above and below, greatly atrophied. An unhealthy-looking sinus, discharging a thin, watery pus, was seen in front about an inch below the acromion. Motions were very limited and painful, but a good deal of looseness and grating of the joint was noticed. Under narcosis, the sinus was enlarged and the finger could then easily be introduced into the joint, where a large sequestrum could be felt. The joint was thereupon resected, Langenbeck's anterior incision being used, and the head of the humerus removed at the surgical neck. The head was found in a state of caries, the cartilage had disappeared, and soft tuberculous-looking granulations covered the denuded surfaces. A large sequestrum was found in the joint, representing the whole glenoid process of the scapula. The osteitic process had evidently commenced here and then invaded the whole joint, just as would have happened in case number six, if it had not been discovered and promptly operated. The diseased synovial capsule was thereafter removed. The long head of the biceps had completely disappeared. The long anterior incision was closed by sutures, and a drainage-tube inserted through an incision at the lowest posterior angle. The wound healed by first intention, the tube was removed on July 10th, and the old patient left the hospital, on July 18th, in good health and with a quite useful arm.

November 20, 1893, she had gained twenty-two pounds in weight, feels well and is able to use her arm in her daily work. A small fistula is still left through which occasionally a little pus is discharged.

### TUBERCULOUS ARTHROITIS OF ELBOW-JOINT—RESECTION.

Case VIII.—Andrew Steislinger, aged 25, horseshoer, entered the hospital on June 17, 1893, for the fourth time in two and one-half years.

The first time, in February, 1891, he had suffered for six months with pain, stiffness, and swelling of left elbow-joint, and had been treated for rheumatism. A chronic synovitis, without bone affection, was diagnosticated. Three large moxies were applied with the thermo-cautery posteriorly, and a plaster-of-Paris dressing applied. He was discharged in a few weeks with a normal joint and without pain.

He reëntered in July, 1892, with an abscess over the olecranon. It was found to be dependent on a tuberculous focus in the olecranon, which was promptly removed, and he left the hospital shortly after with a normal joint again.

He entered the hospital again in February, 1893. At that time the whole joint was swollen, lateral motion, crepitation, and pain on crowding the joint surfaces together were present. The old focus in the olecranon seemed well, and as no other could be discovered, he was considered to suffer from a tuberculous synovitis, pure and simple. Injections of iodoform and glycerine (twenty per cent.) were used, and he improved quite remarkably for a time, although he was not perfectly recovered when he left the hospital. In April a fistula formed, which had discharged since. I did not see him again until he entered the hospital on June 17, 1893, in a much worse condition. The elbow-joint was greatly swollen, with muscular atrophy above and below, great lateral mobility with crepitation, intense pain by crowding the joint surfaces together. The joint was flexed to an angle of 120 degrees, and scarcely any flexion was possible. Two sinuses were found, one leading down to the head of the radius, another into the old focus in the olecranon.

Under narcosis, resection of the elbow was performed by posterior median incision and subperiostally. The humerus was sawn off immediately above the condyles, the bones of the forearm just beneath the head of the radius. The whole capsule was removed, the cavity packed with iodoform gauze, the wound sutured except in the center, and an antiseptic dressing applied, and over that a plaster-of-Paris dressing, the arm being secured in a right angle. By examination of the removed epiphysis, another local focus was found in the internal condyle of the humerus. It had perforated into the joint and done the damage. June 22d, wound dressed, gauze removed.

June 27th, wound almost healed. Splints with hinges applied and surrounded with plaster-of-Paris dressing, passive motions commenced, and patient discharged to his home.

October 25, 1893, the final result is very excellent. He can actively flex and extend his arm to almost normal, pronation and supination normal, very little lateral motion in the joint. He has taken up his work of shoeing horses and is able to work well with his resected arm. As a matter of safety, he still uses an apparatus with two lateral hinged splints.

